# In Silico Characterization of Resistance and Virulence Genes in *Aeromonas jandaei* Strains Isolated from *Oreochromis niloticus* in Brazil

**DOI:** 10.3390/microorganisms13051094

**Published:** 2025-05-08

**Authors:** Marcela Laryssa Oliveira Duarte, Diego Lucas Neres Rodrigues, Gabryel Bernardo Vieira de Lima, Juan Carlos Ariute, Gisele Veneroni Gouveia, João José de Simoni Gouveia, Vasco Azevedo, Bertram Brenig, Eric Guédon, Guilherme Campos Tavares, Mateus Matiuzzi da Costa, Ulisses de Pádua Pereira, Flávia Figueira Aburjaile

**Affiliations:** 1 Preventive Veterinary Medicine Department, Federal University of Minas Gerais, Belo Horizonte 31270-901, MG, Brazil; mduarte.md@gmail.com (M.L.O.D.); diego.neresr@gmail.com (D.L.N.R.); gb.gabryelbernardo@gmail.com (G.B.V.d.L.); gcamposvet@hotmail.com (G.C.T.); 2 Campus of Agrarian Sciences, Federal University of the São Francisco Valley, Petrolina 56300-000, PE, Brazil; gisele.veneroni@univasf.edu.br (G.V.G.); joao.gouveia@univasf.edu.br (J.J.d.S.G.); mateus.matiuzzi@gmail.com (M.M.d.C.); 3 Genetics, Ecology and Evolution Department, Federal University of Minas Gerais, Belo Horizonte 31270-901, MG, Brazil; vascoariston@gmail.com; 4 Institute of Veterinary Medicine, University of Göttingen, 37073 Göttingen, Germany; bbrenig@gwdg.de; 5 STLO, INRAE, Institut Agro, 35011 Rennes, France; eric.guedon@inrae.fr; 6 Preventive Veterinary Medicine Department, State University of Londrina, Londrina 86057-970, PR, Brazil; upaduapereira@gmail.com

**Keywords:** aquaculture, bioinformatics, fish diseases, pathogenomics, resistome

## Abstract

Understanding the genetic characteristics of *Aeromonas jandaei* in Brazilian aquaculture is crucial for developing effective control strategies against this fish pathogen. The present study conducted a genomic analysis of Brazilian *A. jandaei* strains with the objective of investigating their virulence potential and resistance profiles. Four Brazilian isolates were subjected to sequencing, and comparative genomic analyses were conducted in conjunction with 48 publicly available *A. jandaei* genomes. The methods employed included quality assessment, de novo assembly, annotation, and analyses of antimicrobial resistance and virulence factors. The results demonstrated the presence of fluoroquinolone resistance genes within the core genome. Notably, these antibiotics are not authorized for use in aquaculture in Brazil, suggesting that their resistance determinants may originate from other selective pressures or horizontal gene transfer unrelated to aquaculture practices. The analysis identified significant virulence mechanisms, including T2SS, T3SS, and notably T6SS (*vgrG3* gene), which was more prevalent in Brazilian isolates. Additionally, genes associated with motility, adhesion, and heavy metal resistance were identified. These findings highlight the enhanced adaptability of Brazilian *A. jandaei* strains and raise concerns about antimicrobial resistance in aquaculture, emphasizing the need for improved regulatory oversight and control strategies.

## 1. Introduction

Aquaculture has witnessed a significant expansion in recent decades, becoming an increasingly crucial aspect of global food security and economic development. In 2022, aquaculture surpassed capture fisheries in terms of aquatic animal production for the first time, reaching 94.4 million tonnes and accounting for 51% of the global total [1]. However, intensive production faces significant challenges, particularly disease outbreaks caused by high-density and stressful conditions, which frequently result in substantial economic losses [2]. Obtaining accurate data on Brazilian fish production, disease outbreaks, and associated losses remains challenging. However, an estimate suggests that direct and indirect losses amount to approximately USD$ 84 million annually [3].

Among the bacterial pathogens affecting aquaculture, the genus *Aeromonas*, particularly *Aeromonas jandaei*, has emerged as a significant concern in freshwater environments, particularly affecting Nile tilapia (*Oreochromis niloticus*) [4]. This genus consists of Gram-negative, facultative anaerobic bacteria widely distributed in aquatic ecosystems. Phenotypic identification methods often fail to differentiate between species due to the complexity of the genus’s taxonomy [5]. In fish, Aeromonas infections, particularly Motile Aeromonad Septicaemia (MAS), are among the most frequent bacterial diseases, especially in freshwater species. Stress or immune suppression increases susceptibility to MAS, which presents with non-specific clinical signs such as fin rot, ulcers, hemorrhages, exophthalmia, and ascitis [6].

*A. jandaei* has also been reported in Brazilian native species such as pirarucu (*Araipamas gigas*) and tambaqui (*Colossoma macropomum*) [7,8,9], and also in walking catfish (*Clarias sp.*) [10], European eel [11], freshwater crocodiles [12], as well as soft-shell turtles [13]. Its virulence is driven by multiple factors, including haemolysin, temperature-sensitive protease, haemolysin-aerolysin, and nuclease, which facilitate tissue invasion and immune evasion [4]. If not promptly and adequately treated, *Aeromonas* infections can result in significant mortality among affected fish populations [14].

The emergence of antimicrobial resistance in aquaculture pathogens, including *Aeromonas* spp., has become a growing concern globally. This situation is aggravated by the limited number of approved antibiotics for aquaculture use [15]. Understanding the genetic basis of virulence and antimicrobial resistance in these pathogens has become crucial for developing effective control strategies. Recent years have witnessed a shift in understanding bacterial pathogens through genomic approaches. Bioinformatics and genomic analyses have become essential tools for characterizing virulence factors, antimicrobial resistance mechanisms, and evolutionary relationships among bacterial strains. In Brazil, where aquaculture represents a growing sector of the economy, understanding the genetic characteristics of local *A. jandaei* isolates becomes crucial for developing effective control strategies. This study employs comprehensive genomic analysis to investigate Brazilian *A. jandaei* strains, aiming to provide insights into their virulence potential and resistance profiles, contributing to the broader understanding of this important fish pathogen.

## 2. Materials and Methods

### 2.1. Isolation and Genome Sequencing

Four isolates were obtained from Nile tilapia (*Oreochromis niloticus*) exhibiting signs of disease from the São Francisco River in the northeast of Brazil. Two of the isolates (designated Aer_On4M and Aer_On5M) were obtained in 2009 in Sobradinho, in the state of Bahia. The remaining two isolates (GTBM29 and GT15) were derived from fish collected in Petrolina, Pernambuco, and were obtained in 2011. They were isolated from the kidneys of different fish.

Isolates were grown in Tryptic Soy Agar (TSA) at 28 °C for 24 h. All isolates were stored in brain-heart infusion (BHI) broth supplemented with glycerol at −80 °C until analysis. The DNA was extracted from the samples using a salting-out method [16]. Sequencing was conducted on the Illumina Hi-Seq 2500 platform (Illumina, San Diego, CA, USA) using a 2 × 150 bp paired-end library (500 bp insert) at the University of Göttingen, Germany.

### 2.2. Quality Control, Assembly, Completeness, and Annotation

The sequencing quality of the samples was evaluated using FastQC (v0.11.2) metrics [17]. The adapters were removed using Trimmomatic (v0.39) [18].

Subsequently, Unicycler (v0.4.7) [19] was employed for assembly using the default parameters for the de novo method. The quality of the assembled genomes was verified using QUAST (v5.0.2) [20] with the default parameters. To ascertain the completeness of all genomes, a BUSCO (Benchmarking Universal Single-Copy Orthologs) (v4.1.2) [21] analysis was conducted using the gammaproteobacteria_odb10 database and a minimum completeness threshold of 95%. Prokka (1.14.6) [22] was employed for both functional and structural annotation of all genomes to achieve better standardization.

### 2.3. Data Acquisition

All the 48 *A. jandaei* genomes available at the public genome repository of the National Center for Biotechnology (NCBI) [23] were acquired in .fna files to complete this study database on 5 August 2024. All genomes were classified by strain, size (mb), assembly, and host/isolation source (Table 1).

### 2.4. Species Classification

Following this, an Average Nucleotide Identity (ANI) analysis was conducted on all genomes, including the four initial ones and the 48 obtained from NCBI, using the MUMmer alignment method with pyANI (0.2.12) [24]. A similarity threshold of 96% or greater [5] was employed to ascertain whether the genomes in question belonged to the same species.

The phylogenomic tree was constructed using Orthofinder (v2.5.5) [25]. An alignment was conducted with DIAMOND [26] (e-value 5 × 10^−6^), followed by a multiple sequence alignment (MSA). For the alignment of sequences after the identification of clusters of orthologous genes, MAFFT [27] was employed. The phylogenetic tree was constructed using FastTree, and the resulting tree was visualized and edited using iTOL (v7.1) [28].

### 2.5. Resistance and Virulence Genes

The identification of resistance and virulence genes was conducted using PanViTa (v1.1.8) [29]. Genes exhibiting over 70% identity compared to reference sequences were classified as present. The analysis involved comparisons with three databases: for resistance genes, the Comprehensive Antibiotic Resistance Database (CARD) [30] was utilized, while for virulence genes, the Virulence Factors of Pathogenic Bacteria (VFDB) [31], and finally for metal resistance, the Bacterial Metal Resistance Database (BacMet) [32] were employed.

## 3. Results

### 3.1. Genomic Metrics

The initial quality control assessment demonstrated that all genomes exhibited a PHRED quality score of 30 or higher. The BUSCO analysis established a minimum completeness threshold of 95%. Six strains (SEL_ont, SRR12456170_bin.18_metaWRAP_v1.3_MAG, 3299, 3348, Colony25, and Colony119) failed to reach this threshold and were consequently excluded from subsequent analyses. All study subjects achieved a completeness score of 99.7%. Additionally, adapter sequences were removed from all samples. The assembly of four genomes (GTBM29, GT15, On4M, and On5M) showed the results presented in Table 2.

These genomes are available on GenBank/NCBI, and their BioProject and BioSample are shown in Table 3.

### 3.2. Genome Nucleotide Similarity

**Figure 1:**
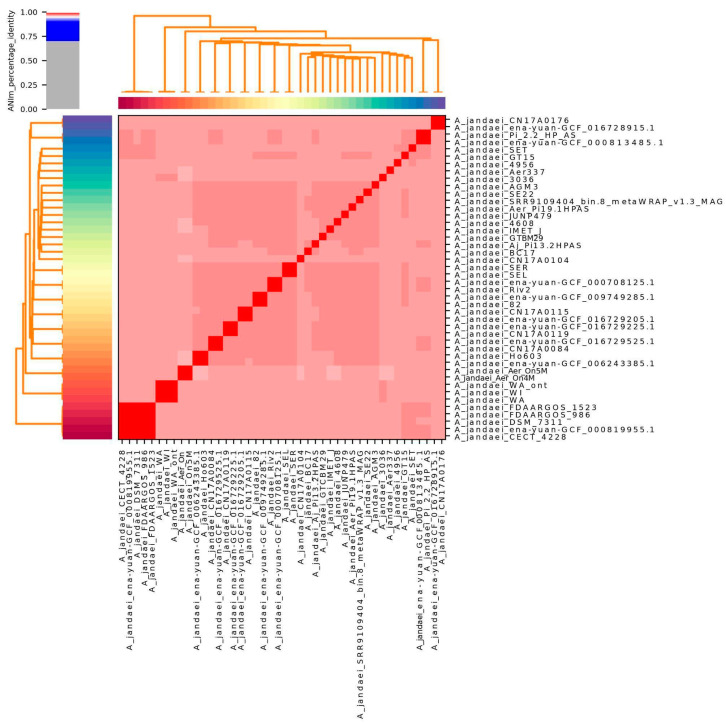
Average Nucleotide Identity (ANI) analysis of 42 *Aeromonas jandaei* genomes from NCBI and 4 from this study. Heatmap shows high genomic similarity (>96%), indicating they belong to the same species.

The average nucleotide identity analysis of 46 strains revealed that 45 isolates exhibited similarity values exceeding 96%, as shown in Figure 1. Two strains (L14h and ena-yuan-GCF_019348715.1) were omitted from the visualization due to lower similarity values. This analysis confirmed that our samples are indeed from *Aeromonas jandaei*. Supplementary Table S1 reveals strain similarities, demonstrating that the two Sobradinho strains (Aer_On4M and Aer_On5M) exhibit high genetic proximity, although they cannot be classified as clonal.

### 3.3. Phylogenomic Analysis of Orthologous Genes

**Figure 2:**
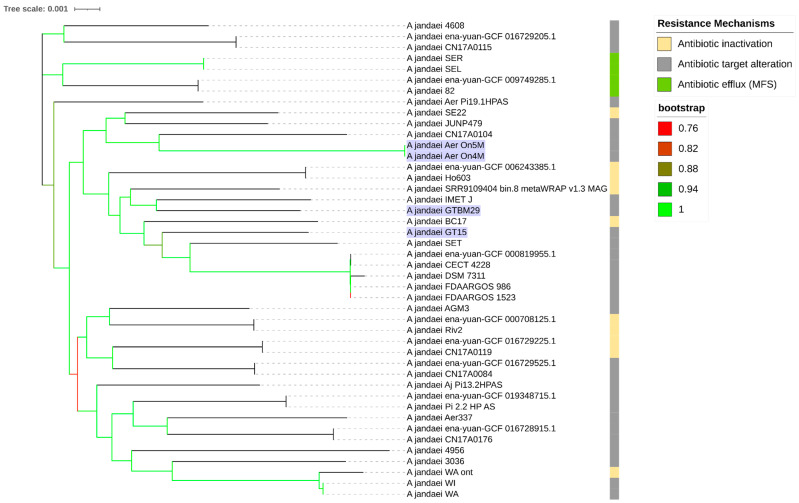
Phylogenomic tree of Aeromonas strains constructed using OrthoFinder. The tree includes 41 genomes from public databases and 4 genomes from the current study (shown in purple). The bootstrap values, which indicate the statistical support for each clade, are displayed next to the corresponding branches. The most prevalent resistance mechanisms for each isolate are shown alongside their respective branches, with color-coding indicating different resistance classes.

The phylogenomic tree of orthologous genes is shown in Figure 2. The tree demonstrates the genetic relationships among *A. jandaei* strains, including our four Brazilian isolates from this study: GT15 and GTBM29 (Petrolina isolates) form a distinct clade with high bootstrap support values, suggesting their close evolutionary relationship, which aligns with their temporal and geographical origin. However, On4M and On5M (Sobradinho isolates), despite being isolated from the same geographical region (northeastern Brazil) as shown in Supplementary Figure S1, cluster separately from the Sobradinho isolates, indicating significant genetic diversity among Brazilian *A. jandaei* strains even within the same region. This suggests that geographical proximity does not necessarily reflect phylogenetic relatedness in this species. The presence of *A. veronii* FDAARGOS_632 as an outgroup helps to root the tree and provides context for the evolutionary relationships within *A. jandaei* species. The tree topology suggests significant genetic diversity among *A. jandaei* strains, with several distinct lineages being observed.

To explore potential genomic variation across isolates, we examined the accessory genome composition. To maintain clarity, we present the complete accessory genome matrix, showing presence/absence patterns for all accessory genes, as Supplementary Figure S2.

### 3.4. Resistance Genes

#### 3.4.1. Antimicrobial Resistance Results

The CARD analysis revealed a total of 28 genes. Five genes were found on the core genome (bacA, OXA-12, MCR-7.1, CRP, and rsmA). Genes bacA and MCR-7.1 are involved in antibiotic target alteration and confer resistance to peptide antibiotics, as well as the OXA-12 gene responsible for antibiotic inactivation and providing resistance to penam antibiotics. Additionally, the strains with the CRP and rsmA genes are associated with antibiotic efflux and mediate resistance to a broader range of antibiotics, including penams, fluoroquinolones, macrolides, phenicols, and diaminopyrimidines.

The accessory genome contained 19 antibiotic resistance genes with diverse functions. This included 9 genes involved in antibiotic target alteration, such as the *MCR-3* family (*MCR-3.1*, *MCR-3.28*, *MCR-3.37*, *MCR-3.9*, *MCR-3.2*, *MCR-3.6*, *MCR-3.36*, and *MCR-3.33*) and *arnA* conferring resistance to peptide antibiotics, as well as 7 genes responsible for antibiotic inactivation, including *cphA3* and *cphA7* targeting carbapenems, *AQU-1*, *AQU-2*, and *AQU-3* active against cephalosporins, *FOX-2* effective against cephamycins and cephalosporins, and *TRU-1* providing resistance to penams and cephalosporins. Additionally, 2 genes associated with efflux mechanisms, *tet (E)* and *tet (A)*, were identified, providing resistance to tetracycline antibiotics. The accessory genome also had 1 gene, *QnrS6*, linked to protection of the antibiotic target and conferring resistance to fluoroquinolones.

In addition, 4 exclusive genes were identified: *FOX-15*, a gene for antibiotic inactivation effective against cephamycins and cephalosporins; *ugd*, a gene for antibiotic target alteration conferring resistance to peptide antibiotics; *acrB*, a gene for antibiotic efflux providing resistance to tetracyclines, disinfecting agents and antiseptics, phenicols, rifamycins, penams, glycylcyclines, cephalosporins, and fluoroquinolones; and *PmrF*, a gene for antibiotic target alteration also associated with peptide antibiotic resistance.

**Figure 3:**
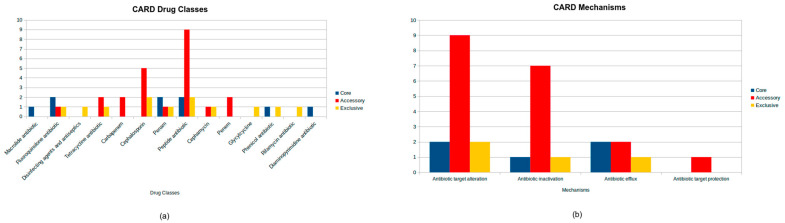
CARD database antimicrobial resistance analysis in *Aeromonas jandaei*, showing the distribution of drug classes (**a**) and antimicrobial resistance mechanisms (**b**), highlighting the number of genes belonging to core, accessory, and exclusive genomes.

The drug classes and mechanisms related to all strains can be seen in Figure 3.

The PanViTa analysis (Supplementary Figure S3) shows random distribution of accessory AMR genes among the strains, with no shared resistance patterns. Most resistance genes were located in the core genome, while the few accessory AMR genes displayed strain-specific occurrences. When looking exclusively into the four Brazilian strains from this study, the gene distribution can be seen in Table 4.

#### 3.4.2. Heavy Metal Resistance Results

**Figure 4:**
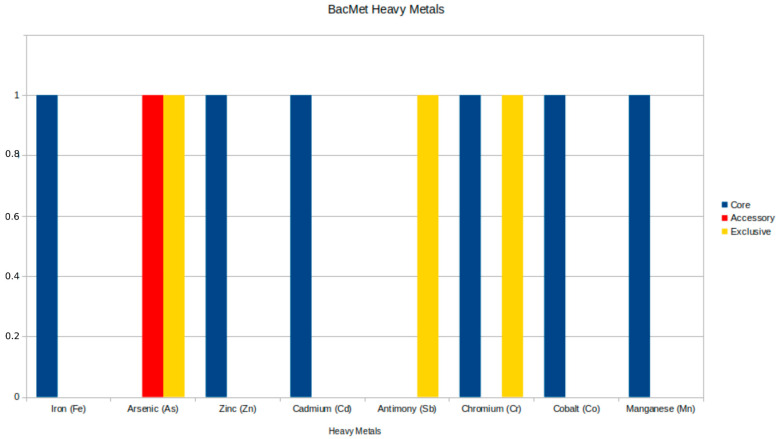
Genomic distribution of heavy metal resistance genes from the BacMet database in *Aeromonas jandaei*, illustrating the prevalence and diversity of resistance mechanisms across different genome categories.

The analysis of resistance to heavy metals revealed five significant genes: two in the core genome and one in the accessory genome, as detailed in Table 5, along with two exclusive genes (*arsA* and *recG*) found in other genomes beyond the Brazilian samples. The core genome genes include the *ruvB* gene, which encodes an ATP-dependent DNA helicase from the Holliday junction and is associated with resistance to chromium (Cr), and the divalent cation transporter *mntH/yfeP*, which shows resistance to multiple metals, including manganese (Mn), iron (Fe), cadmium (Cd), cobalt (Co), and zinc (Zn). The *arsC* gene, an accessory gene, encodes the ArsC protein and is specifically related to resistance to arsenic (As). The distribution of heavy metal resistance genes is illustrated in Figure 4. Supplementary Figure S4 illustrates the distribution of genes across all strains, revealing that the majority of lineages, including the strains from this work, do not have accessory genes.

### 3.5. Virulence Genes

**Figure 5:**
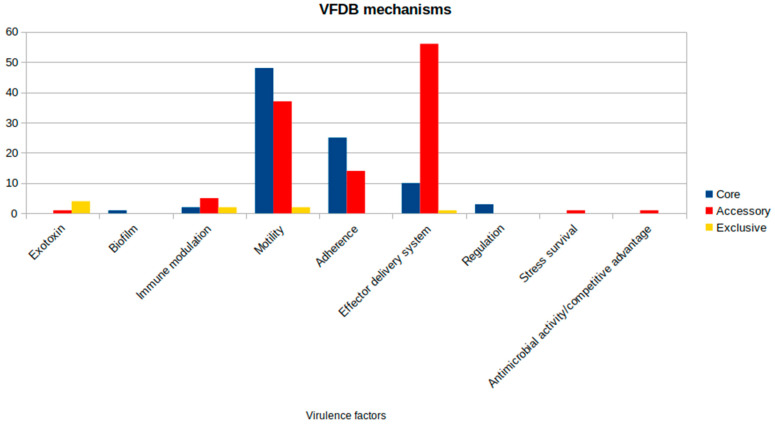
Virulence factors distribution by class from the Virulence Factor Database (VFDB) in *Aeromonas jandaei*.

The virulence factors analysis revealed an amount of 213 genes present within the genetic profile of the subjected strains. Amongst this set, 89 genes are shared by all analyzed strains. Most of the core gene sets are related to motility (48) and adhesion (25) mechanisms. A less obvious portion is linked to the well-described effector delivery system (10). However, when looking at genes related to the accessory compartment, a greater prevalence of this class of virulence factors is highlighted, as shown in Figure 5. Considering this result, there are some attention-grabbing outcomes that could lead to a new hypothesis regarding the pathogenicity of the species. All identified genes and their associated virulence mechanisms are detailed in Supplementary Table S2. Their distribution across strains (Supplementary Figure S5) reveals a consistent pattern distinguishing the On4M and On5M isolate groups, with shared virulence profiles within each cluster that align with their phylogenetic relationships.

Regarding the highlighted Brazilian strains, a set composed of a few genes is intriguing. Five genes (*rfbF*, *tppE*, *tppB*, *tapY1,* and *tppA*) are shared by two out of four genomes that were isolated from the same geographical localization. In this context, four genes (*tppE*, *tppB*, *tapY1,* and *tppA*) are linked to adhesion processes through pilus formation, and one gene is related to immunomodulation (*rfbF*). On the other hand, the *vgrG3* gene is present in all subjects and is more prevalent in Brazilian strains, considering isolates other than that directly related to this study.

## 4. Discussion

### 4.1. Resistome Data

It is essential to start pointing to the core genes that were found, as seen in Figure 3. There are three different classes showing a major presence in the core genomes: the fluoroquinolones, penams, and peptide antibiotics. The fluoroquinolones are interesting because of the fact that a previous study identified this class as very relevant in in vitro essays, exhibiting its high susceptibility when used against *Aeromonas* spp. isolated from fish [33]. However, a previous study [34] says otherwise: when a variety of *Aeromonas* were submitted to an antimicrobial test, they expressed a higher resistance than the previous study (>60%) to nalidixic acid, a fluoroquinolone. Changing to the genotypic characteristics, a work has done the resistome identification of five species of the *Aeromonas* genus, showing that the resistance genes for fluoroquinolones were very equally distributed among all of the five species [35]. In this way, it is possible to understand that it is not very surprising that these genes for fluoroquinolones are in the core genomes of the *Aeromonas jandaei* of this study, but it is still worrying for the fact that these kinds of antimicrobials are more recent and already have so many resistance genes distributed among the *Aeromonas* species that should be more rare [36].

Another concerning aspect regarding fluoroquinolones in Brazil is their current unregulated status in aquaculture. Fluoroquinolones are already approved for aquaculture in many other major producing countries, including the European Union, China, Japan, and Thailand. While some fluoroquinolones are regulated in Brazil for use in other livestock species, their use in aquaculture remains unregulated [37]. This regulatory gap is particularly worrying given that residual enrofloxacin has been detected in *O. niloticus* (tilapia) fillets [38], which indicates its illegal use [39,40] since only oxytetracycline [41] and florfenicol [42] are currently authorized. Furthermore, the bacterial isolates from Brazilian aquaculture in this study have demonstrated a possible resistance to fluoroquinolones, even though these antimicrobials are not officially approved for aquaculture use.

Resistance to fluoroquinolones is often linked to specific mutations in the *gyrA* and *parC* genes. These mutations have been well-documented in many bacterial species and are known to play a major role in fluoroquinolone resistance [43,44]. To check if our four *A. jandaei* isolates carried similar mutations, we analyzed the *gyrA* and *parC* genes using MEGAres, a database designed to detect antimicrobial resistance genes [45]. Our analysis showed no signs of the typical fluoroquinolone-resistance mutations found in other bacteria. This finding matches our earlier observation that fluoroquinolone resistance genes were missing from the accessory genomes of these isolates. The lack of these mutations suggests that changes in *gyrA* and *parC* are unlikely to be responsible for fluoroquinolone resistance in our *A. jandaei* isolates. Further research is needed to explore other potential mechanisms behind this resistance. 

For penam resistance, a Brazilian study has used nine *Aeromonas veronii* from animal (fish), human, and environmental sources and some NCBI public genomes for the identification of resistance genes from different classes of antimicrobials. As a result, they have found that the resistance genes from penam were not so abundant; the majority of the isolates and the NCBI ones had only two genes linked to the penam, except one from NCBI that had 15 [46]. Other genotypic analyses, the previous work [47] that used 26 isolates of various species, 13 of them *Aeromonas*, that were isolated from different fishes and water sources, exhibit in PCR that genes for penam resistance were the second most present among the isolates, that gene being a representative from the *bla* family. Furthermore, a study on *A.veronii* isolated from a fish in comparison with other complete genomes from the *Aeromonas* genus has shown that the gene *OXA-12*, a gene from penam resistance, was one of the most present in the results, which is quite normal in *Aeromonas* spp. and has also been present in *A. jandaei* [48,49].

The last major core resistance class was peptide antibiotics, which in this case are defined for the genes from the *MCR* family, *MCR-7.1* and *MCR-3.1*. Both of them are linked to the resistance to colistin; they are also found in the most diverse types of bacterial species and can originate or serve as a reservoir of these types of resistance in the environment or other ambients they are in [50].

### 4.2. Heavy Metal Resistance

Heavy metal resistance in *Aeromonas* has been documented in the literature, revealing variations among different studies. In our research, we identified resistance to several heavy metals, including chromium (Cr), arsenic (As), manganese (Mn), iron (Fe), cadmium (Cd), cobalt (Co), and zinc (Zn), alongside the identification of three resistance genes: *ruvB*, *arsC,* and *mntH/yfeP*.

Comparing these findings with those from other studies, it can be observed that cadmium often exhibits high resistance rates, ranging from 9.1% to 61% in isolates [51,52], while resistance to chromium is also significant, with up to 95.3% of isolates showing resistance [51]. Although zinc showed high resistance in some isolates (67.3% to 100%) [52,53], our data complement this information by indicating resistance to other metals, such as arsenic, which were not highlighted in previous studies. In addition, the genes identified in our study, such as *arsC* and *mntH/yfeP*, expand the understanding of the molecular mechanisms underlying heavy metal resistance, which in the literature also includes genes such as *copA* and *czcA* [52]. The differences in resistance levels and the presence of resistance genes reflect the complexity of the adaptation of *Aeromonas* to polluted habitats and suggest that further research is needed to understand the relationship between heavy metal resistance and environmental contamination [54].

### 4.3. Virulence Factors

The genetic profile analyzed offers a perspective on the virulence factors of *Aeromonas jandaei*. Focusing on specific factors, there is a significant relevance of elements related to motility and adhesion, such as the genes *fimA*, *fimC*, *fimD*, *fimE*, and *fimF*, which are associated with pili formation and host adhesion [55]. These genes are essential for free-living pathogens such as *Aeromonas* species [56,57], since host adhesion is crucial for the start of tissue colonization and enables bacterial cells to be protected from physical and chemical challenges through the formation of biofilm structures [57,58].

The isolates analyzed also exhibited a gene complex composed of *flg* and *flr* genes, which are linked to flagellar biosynthesis [59,60]. The importance of flagella for motility in the environment is mainly relevant for free-living pathogens, allowing them to migrate to a more suitable location and enhance surface contact, making this adaptation a significant evolutionary acquisition [59].

Another critical point is the presence of elements related to secretion systems. Genes associated with type II (T2SS) and type III (T3SS) secretion systems are fundamental to bacterial pathogenicity [61,62]. These systems allow the bacteria to release enzymes and toxins into the extracellular environment or directly into the cytoplasm of the host cells, promoting invasion and causing significant tissue damage [63].

An interesting finding is the higher prevalence of the *vgrG3* gene in Brazilian isolates. It is a part of the type VI secretion system (T6SS), and this system is known for its aggressive capability, acting as a molecular harpoon that injects toxins and effectors directly into the cytoplasm of the target cells [64,65]. Beyond its role in pathogenicity, the T6SS is an important factor in bacterial competition [66], which in theory would enable *Aeromonas jandaei* to eliminate other competing bacteria, particularly in aquatic environments.

The presence of these genomic mechanisms suggests an increased competence of *Aeromonas jandaei* strains to colonize and persist in different environments and conditions, particularly in host interactions.

## 5. Conclusions

Genome analysis of *Aeromonas jandaei* isolates from Brazilian aquaculture environments has offered significant insights into the antimicrobial resistance potential, heavy metal resistance, and virulence mechanisms. The investigation of *Aeromonas jandaei* from Brazilian aquaculture environments has revealed the presence of genes related to multiple resistance mechanisms and virulence factors, which may pose a risk to both aquaculture production and public health. The identification of these genetic elements through bioinformatics approaches provides crucial information for the development of more effective disease prevention and control strategies in aquaculture settings.

The findings underscore the necessity for the establishment of comprehensive genomic surveillance programs in Brazilian aquaculture. The regular monitoring of bacterial populations through genomic tools can facilitate the early detection of emerging resistance patterns and potential pathogens, thereby enabling the implementation of effective control measures before they become significant problems. This approach is of particular importance given the economic significance of aquaculture in Brazil and the potential impact of bacterial diseases on production.

To mitigate these risks, it is essential to strengthen sanitary regulations and preventive measures. This includes implementing improved biosecurity protocols, enhancing water quality management, and developing more effective vaccination strategies. Furthermore, the establishment of clear guidelines for antimicrobial use in aquaculture, in conjunction with regular monitoring of bacterial populations, could help prevent the emergence and spread of resistant strains.

## Figures and Tables

**Table 1:** Genomes of *A. jandaei* downloaded from NCBI database.

*A. jandaei*				
Strain	Size (Mb)	Assembly	Host/ Isolation Source	Location
FDAARGOS_986	4.6	GCF_016127195.1	Unknown	Germany: Braunschweig
DSM 7311	4.6	GCF_037890695.1	*Homo sapiens* (stool)	USA: Oregon
3036	4.6	GCF_018802365.1	Chicken	USA: California
JUNP479	4.5	GCF_016865345.1	*Homo sapiens*	Nepal
BC17	4.5	GCF_026651485.1	River water	Ghana
4608	4.5	GCF_018802325.1	Poultry	USA: California
FDAARGOS_1523	4.5	GCF_020341535.1	Unknown	Germany: Braunschweig
Aer_Pi19.1HPAS	4.6	GCF_017310175.1	*Phractocephalus hemioliopterus* (kidney)	Brazil: Petrolina
AGM3	4.4	GCF_036687345.1	*Heteroapneustes fossilis* (lesion)	Bangladesh: Mymensingh
CECT 4228	4.5	GCF_000819955.1	Unknown	Unknown
IMET J	4.7	GCF_002925765.1	Marine water	USA
CN17A0115	4.5	GCF_016729205.1	*Homo sapiens* (stool)	China: Shenzhen, Longgang
ena-yuan-GCF_016729205.1	4.5	GCF_963890675.1	Unknown	Unknown
CN17A0084	4.5	GCF_016729525.1	*Homo sapiens* (stool)	China: Shenzhen, Longgang
ena-yuan-GCF_016729525.1	4.5	GCF_963891405.1	Unknown	Unknown
Pi_2.2_HP_AS	4.6	GCF_019348715.1	*Phractocephalus hemioliopterus* (kidney)	Brazil: Petrolina
ena-yuan-GCF_019348715.1	4.6	GCF_963894795.1	Unknown	Unknown
CN17A0176	4.6	GCF_016728915.1	*Homo sapiens* (stool)	China: Shenzhen, Nanshan
ena-yuan-GCF_016728915.1	4.3	GCF_963892185.1	Unknown	Unknown
82	4.8	GCF_009749285.1	Fish	China: Shenzhen
ena-yuan-GCF_009749285.1	4.8	GCF_963886775.1	Unknown	Unknown
CN17A0104	4.4	GCF_016729325.1	*Homo sapiens* (stool)	China: Shenzhen, Longgang
CN17A0119	4.7	GCF_016729225.1	*Homo sapiens* (stool)	China: Shenzhen, Longgang
ena-yuan-GCF_016729225.1	4.7	GCF_963890895.1	Unknown	Unknown
Aer337	4.7	GCF_003849685.1	*Homo sapiens* (fecal sample)	Brazil: Sao Bento do Una
Aj_Pi13.2HPAS	4.9	GCF_021735865.1	*Phractocephalus hemioliopterus* (kidney)	Brazil: Petrolina
WA	4.6	GCF_034721275.1	Unknown	Unknown
WI	4.6	GCF_034720845.1	Unknown	Unknown
SE22	4.5	GCF_034092435.1	Unknown	Unknown
ena-yuan-GCF_000819955.1	4.5	GCF_963867185.1	Unknown	Unknown
SEL	4.9	GCF_034720715.1	Unknown	Unknown
SET	4.7	GCF_034092485.1	Unknown	Unknown
SER	4.9	GCF_034721165.1	Unknown	Unknown
Ho603	4.6	GCF_006243385.1	*Hirudo orientalis* (gut)	Unknown
ena-yuan-GCF_006243385.1	4.6	GCF_963883215.1	Unknown	Unknown
Riv2	4.5	GCF_000708125.1	River water	USA
ena-yuan-GCF_000708125.1	4.5	GCF_963865165.1	Unknown	Unknown
3299	4.6	GCA_014217505.1	Chicken	USA: California
3348	4.6	GCA_014217485.1	Chicken	USA: California
4956	4.7	GCA_018802265.1	Chicken	USA: California
Colony25	4.6	GCA_016902935.1	Rectal swab	Thailand: Pattani
Colony119	5.1	GCA_016902955.1	Rectal swab	Thailand: Udon Thani
WA_ont	4.7	GCA_036452205.1	Unknown	Unknown
SEL_ont	5	GCA_036452235.1	Unknown	Unknown
SRR9109404_bin.8_metaWRAP_v1.3_MAG	4.4	GCF_945952205.1	Fish gut metagenome	Unknown
L14h	4.7	GCA_000813485.1	Lake water	Malaysia
ena-yuan-GCF_000813485.1	4.7	GCA_963866915.1	Unknown	Unknown
SRR12456170_bin.18_metaWRAP_v1.3_MAG	4	GCA_945957145.1	Fish gut metagenome	Unknown

**Table 2:** Results (bp, contigs, N50, L50, and GC content) obtained on Quast from the four genomes (GTBM29, GT15, On4M, On5M).

Strain	bp	Contigs	N50	L50	GC Content
GTBM29	4681450	64	417315	4	58.75
GT15	4578060	51	200574	8	58.86
On4M	4487199	73	197578	7	59.01
On5M	4488431	74	197525	6	59.01

**Table 3:** BioProjects and BioSamples from GTBM29, GT15, On4M, and On5M.

Strain	BioProject	BioSample
GTBM29	PRJNA590577	SAMN13335576
GT15	PRJNA590949	SAMN13343509
On4M	PRJNA592537	SAMN13428651
On5M	PRJNA607235	SAMN14124565

**Table 4:** Genes found on *Aeromonas jandaei* GT15, GTBM29, Aer_On4M and Aer_On5M strains through CARD analysis.

Gene	Core	Drug Class	Resistance Mechanism	AMR Gene Family
*bacA*	Yes	peptide antibiotic	antibiotic target alteration	undecaprenyl pyrophosphate related proteins
*OXA-12*	Yes	penam	antibiotic inactivation	OXA beta-lactamase
*MCR-7.1*	Yes	peptide antibiotic	antibiotic target alteration	MCR phosphoethanolamine transferase
*CRP*	Yes	penam, fluoroquinolone antibiotic, macrolide antibiotic	antibiotic efflux	resistance-nodulation-cell division (RND) antibiotic efflux pump
*MCR-3.1*	Yes	peptide antibiotic	antibiotic target alteration	MCR phosphoethanolamine transferase
*rsmA*	Yes	phenicol antibiotic, diaminopyrimidine antibiotic, fluoroquinolone antibiotic	antibiotic efflux	resistance-nodulation-cell division (RND) antibiotic efflux pump
*arnA*	Yes	peptide antibiotic	antibiotic target alteration	pmr phosphoethanolamine transferase
*cphA7*	Yes	carbapenem	antibiotic inactivation	CphA beta-lactamase
*FOX-2* *	No	cephamycin, cephalosporin	antibiotic inactivation	FOX beta-lactamase
*AQU-2* **	No	cephalosporin	antibiotic inactivation	AQU beta-lactamase

* Found in GT15, Aer_On4M and Aer_On5M. ** Found in GTBM29.

**Table 5:** Three genes represent all heavy metal resistance genes identified in the four Brazilian genomes analyzed in this study.

Gene	Mechanism	Compound
*ruvB*	Holliday junction ATP-dependent DNA helicase RuvB	Chromium (Cr)
*arsC*	Protein ArsC	Arsenic (As)
*mntH/yfeP*	Divalent metal cation transporter MntH	Manganese (Mn), Iron (Fe), Cadmium (Cd), Cobalt (Co), Zinc (Zn)

## Data Availability

All genomes are available at NCBI—a public database.

## Supplementary Materials

The following supporting information can be downloaded at: https://www.mdpi.com/article/10.3390/microorganisms13051094/s1, Figure S1: Geographic location of bacterial isolation sites in the São Francisco River region; Figure S2: Presence/absence matrix of accessory genes among *Aeromonas jandaei* isolates, alongside a phylogenetic tree; Figure S3: Pangenome analysis revealing gene distribution and identity across bacterial strains; Figure S4: Clustering of heavy metal resistance genes across *Aeromonas* strains; Figure S5: Clustermap of virulence gene presence and identity across *Aeromonas jandaei* strains. Supplementary Table S1: Pairwise genomic similarity analysis of *Aeromonas jandaei* strains. The two Sobradinho isolates (Aer_On4M and Aer_On5M) show high genetic proximity (>99% ANI) yet distinct genomic features that preclude clonal classification, suggesting closely related but independently evolving strains. Values represent average nucleotide identity (ANI) percentages. Supplementary Table S2: Virulence-associated genes and their functional mechanisms.
