# Supplementary figures and images for: In Silico Characterization of Resistance and Virulence Genes in Aeromonas jandaei Strains Isolated from Oreochromis niloticus in Brazil
Source: Microorganisms. 2025 May 8;13(5):1094. doi: 10.3390/microorganisms13051094 (PMC12114387; doi:10.3390/microorganisms13051094)

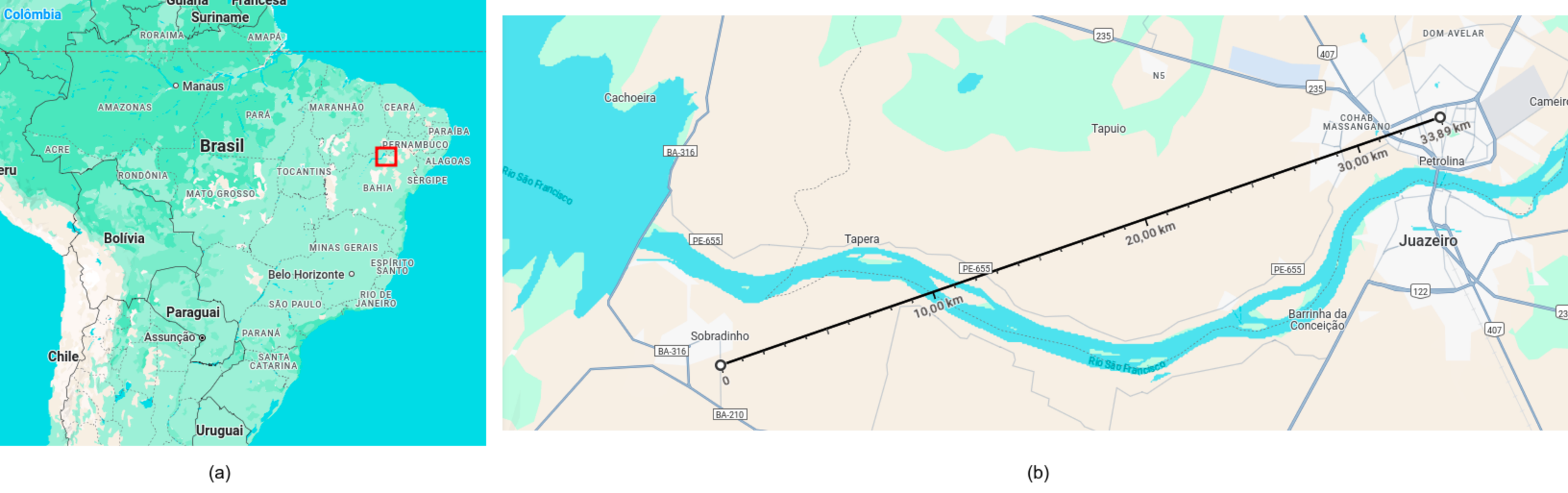

Supplement: Supplementary file 1 [file microorganisms-13-01094-s001.zip › Figure S1.png]

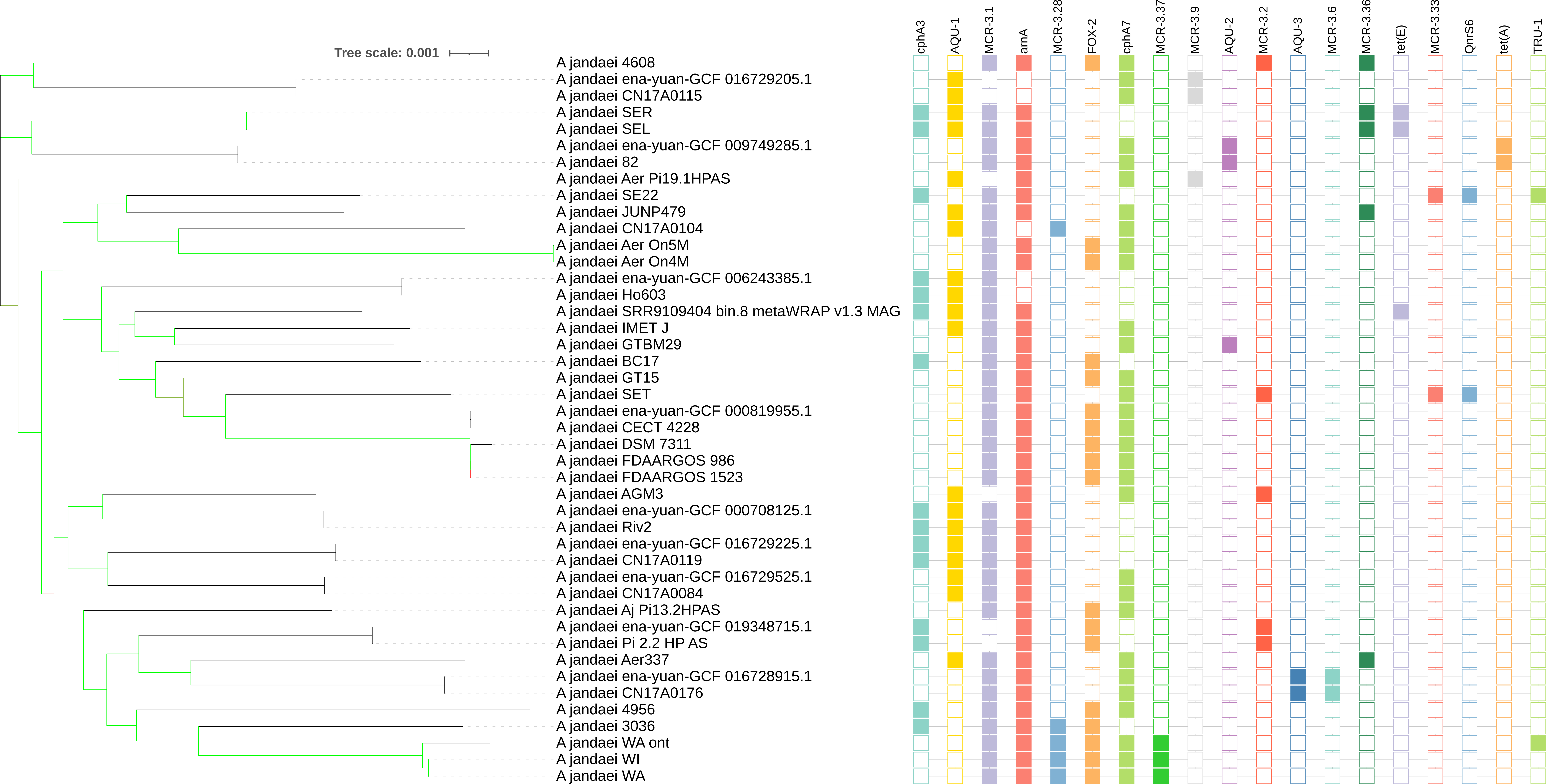

Supplement: Supplementary file 1 [file microorganisms-13-01094-s001.zip › Figure S2.png]

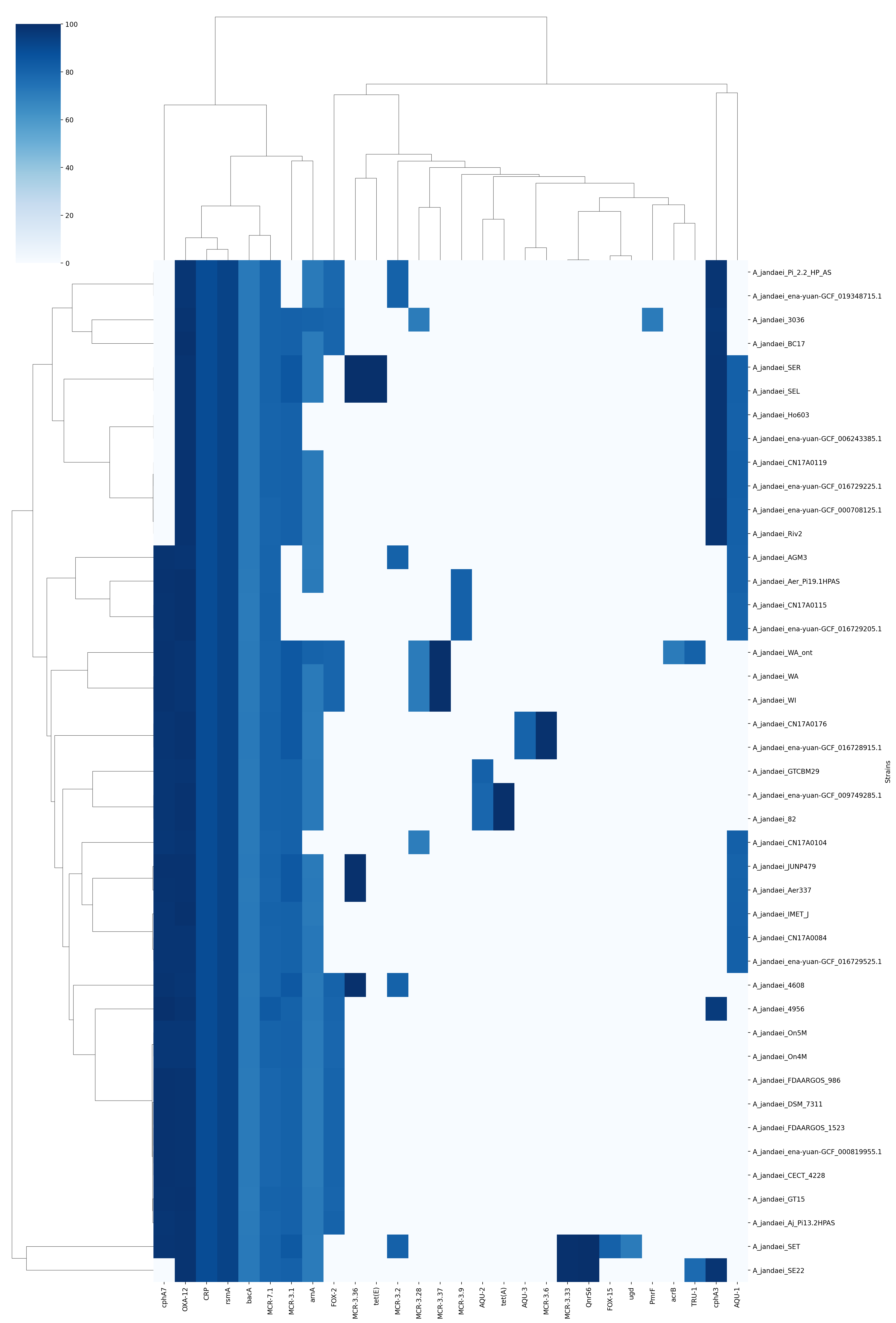

Supplement: Supplementary file 1 [file microorganisms-13-01094-s001.zip › Figure S3.png]

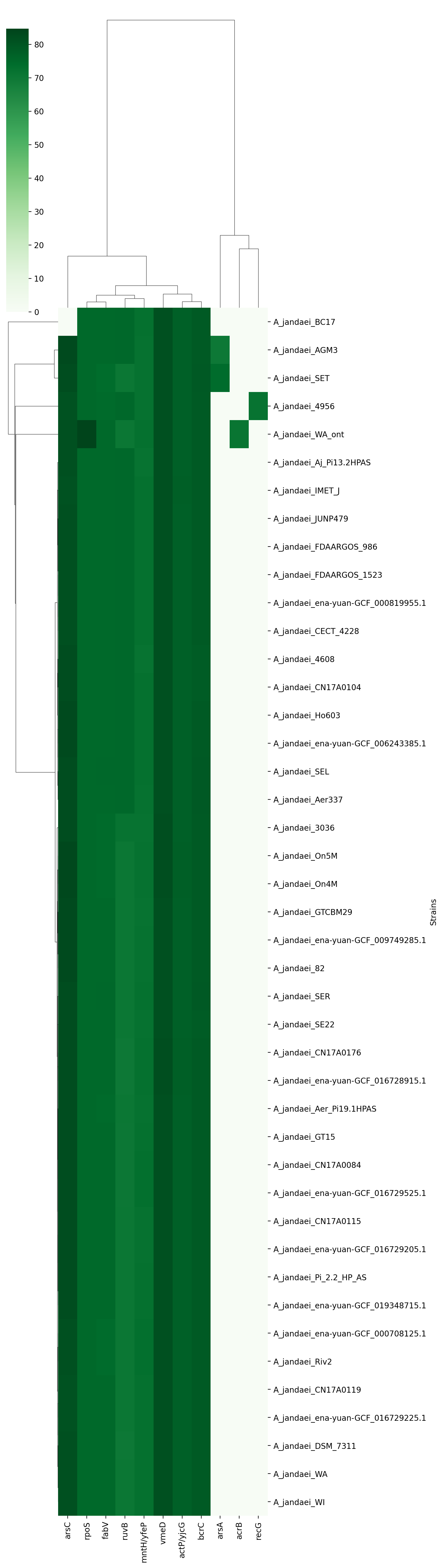

Supplement: Supplementary file 1 [file microorganisms-13-01094-s001.zip › Figure S4.png]
